# Tumor deposits should not be placed in the M category of TNM: A comparative survival analysis using SEER data

**DOI:** 10.1002/ijc.35165

**Published:** 2024-09-05

**Authors:** Ayse Selcen Oguz Erdogan, Femke Simmer, Iris D. Nagtegaal

**Affiliations:** ^1^ Department of Pathology Radboud University Medical Center Nijmegen The Netherlands

**Keywords:** colon cancer, metastasis, SEER, TNM classification, tumor deposits

## Abstract

Tumor deposits (TD) are tumor nodules in the lymphatic drainage area of colorectal cancer patients, and they are currently classified in the N category in the TNM classification. However, due to the associated poor prognosis, some small cohort studies suggest that TD belong in the M category. A retrospective study using The Surveillance, Epidemiology, and End Results program (SEER) data was performed in Stages III and IV colon carcinoma (CC) patients to evaluate the prognostic impact of TD. In multivariate analysis, TD have significantly negative effect on survival in both stages (Stage III HR = 1.4 [95% CI 1.4–1.5] and Stage IV HR = 1.3 [95% CI 1.2–1.3]). In Stage III, 5‐year overall survival (OS) for patients with TD 49%, whereas it was 64% for patients without TD (*p* < .001). Additionally, in Stage IV patients without TD, the 5‐year OS rates are superior at 21% compared to patients with TD, who show 5‐year OS rate of 10% (*p* < .001). Stage III patients with TD (5‐year OS 49%) have a significantly better prognosis compared to Stage IV patients (5‐year OS 17%, *p* < .001). Therefore, despite the previous suggestions, this large scale study (*n* = 52,332) on outcomes in CC does not support the classification of TD in Stage IV.

## Graphical abstract

What's new?

In colorectal cancer (CRC), tumor deposits (TD) that form within the pericolorectal lymph drainage region are classified as N category within the TNM staging system. TD in CRC, however, are associated with poor prognosis and may actually represent distant metastases within Stage IV. Here, the prognostic impact of TD was evaluated in Stage III and Stage IV CRC patients according to survival. Among Stage III patients, 5‐year overall survival was 49% and 64% for those with or without TD, respectively. Relative to Stage IV patients, prognosis was significantly better for Stage III patients, indicating that TD are not distant metastases.

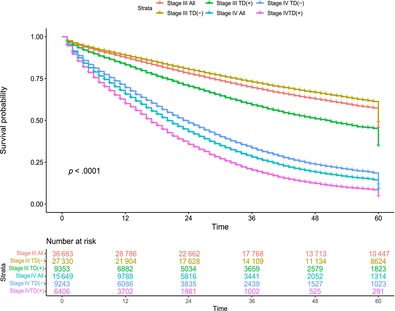

## 1 INTRODUCTION

The spread of tumor cells beyond the primary origin is the main cause of cancer‐related death across various cancer types.^1^ In colorectal carcinoma (CRC) patients, tumor deposits (TD) are a well‐recognized independent poor prognostic marker, which have been defined as discontinuous tumor nodules in the lymphatic drainage area of the pericolorectal region.^2^, ^3^

Although discussions are ongoing about exact definitions and placing, TD are currently staged within the N category of the TNM classification.^2^, ^4^ However, it has been repeatedly suggested that, based on the associated poor prognosis, TD should be classified as distant metastases under the M category.^5^, ^6^, ^7^, ^8^

Therefore, our aim was to compare the survival rates of colon carcinoma patients in Stage III with TD to Stage IV patients in a large cohort and explore whether the prognostic impact of TD is indeed comparable to Stage IV disease. To achieve this, we conducted a retrospective study using the data of The Surveillance, Epidemiology, and End Results (SEER) program.^9^

## 2 MATERIALS AND METHODS

### 2.1 Patient selection

**FIGURE 1:**
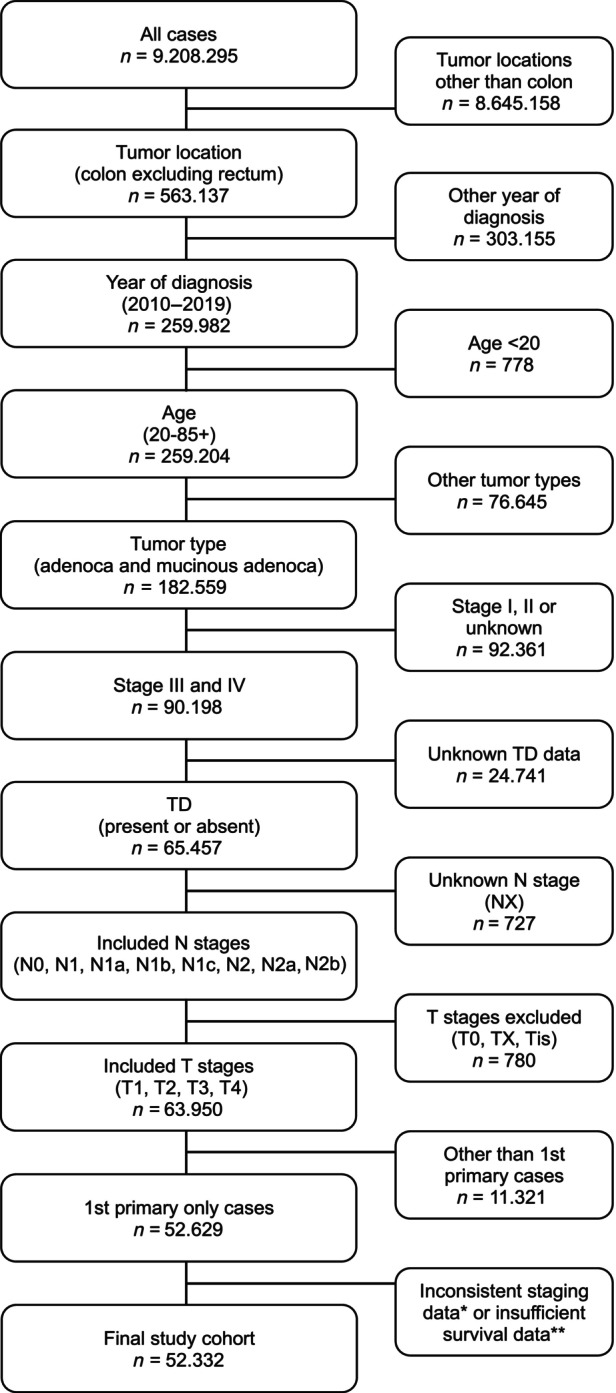
Flowchart of the study cohort selection. TNM stages are based on 7th and 8th edition of TNM *Patients without tumor deposits (TD) and LNM but in Stage III (1), patients with TD but placed in N0 group in Stage IV (253). **Death certificate and autopsy only cases (5), not microscopically confirmed cases (9), alive with no survival time (5), age values not in the expected survival table (24).

The Incidence‐SEER Research Data 17 registries, November 2022 Sub (2000–2020) database of SEER program was used for this research.^10^ The study cohort was limited to patients diagnosed with Stages III and IV colon carcinoma with the tumor types adenocarcinoma, nos (8140/3) and its most common subtype mucinous adenocarcinoma (8480/3), from 2010 onwards. The TNM, T, and N stages were determined by selecting options from the case selection section, which included the Stage‐7th edition (2010–2015), Stage‐7th edition (2016–2017), and Stage‐8th edition (2018+). For survival calculation study cut off was set at the “December 2019” to prevent potential Coronavirus Disease 2019 (COVID‐19) effects on survival. Patients with undocumented/not assessed TD information were excluded from the study and only cases with or without TD, regardless of the number, were included. The right colon was defined from the cecum to transverse colon, while the left colon included the splenic flexura, descending colon and sigmoid. Caselisting data with the defined parameters and observed survival rates were obtained using SEER*stat version 8.4.2 software. We outlined the patient selection process in the Figure 1.

### 2.2 Statistical analysis

The Chi‐squared test was used to assess the associations between patients' clinicopathological features and the presence of TD at different stages.

Observed survival, defined as the time from the date of diagnosis to death for any reason (censored with the study cutoff date), was obtained. Overall survival (OS) curves were generated using the Kaplan–Meier method and group comparisons were conducted by log‐rank test. Univariate and multivariate analyses were carried out using Cox regression analysis. All analyses were performed using SPSS software (SPSS, Chicago, IL) and R (version 2022.02.1). The *p*‐values below .05 were regarded as statistically significant.

## 3 RESULTS

A total of 52.332 patients were included in the study cohort, with 36.683 patients classified as Stage III and 15.649 patients classified as Stage IV. Clinicopathological characteristics of the study cohort were presented in Table 1. In Stage III, the presence of TD was associated with tumor location, T and N category. There was a small effect on delivery of chemotherapy, with less treatment in Stage III with TD (64.1% vs. 65.4%, *p* = .027). In Stage IV there was no effect on treatment. The presence of TD was associated with age, tumor location, tumor type, T and N category.

**TABLE 1:** Clinicopathological features of the study cohort.

	Stage III all cohort *n* = 36,683			Stage IV all cohort *n* = 15,649		
	TD present *n* = 9353 (25.5%)	TD absent *n* = 27,330 (74.5%)	*p*	TD present *n* = 6406 (41%)	TD absent *n* = 9243 (59%)	*p*
Sex						
Female	4677 (50)	13,851 (50.7)		3168 (49.5)	4434 (48)	
Male	4676 (50)	13,479 (49.3)	.260	3238 (50.5)	4809 (52)	.068
Age						
≤65	5145 (55)	14,877 (54.4)		2883 (45)	4409 (47.7)	
>65	4208 (45)	12,453 (45.6)	.336	3523 (55)	4834 (52.3)	<.001
Tumor site						
Right colon	5044 (53.9)	15,974 (58.4)		3638 (56.8)	5006 (54.2)	
Left colon	4085 (43.7)	10,806 (39.5)		2565 (40)	4014 (43.4)	
Large intestine	224 (2.4)	550 (2)	<.001	202 (3.2)	223 (2.4)	<.001
Tumor type						
Adenocarcinoma, nos	8497 (90.8)	24,750 (90.6)		5652 (88.2)	8477 (91.7)	
Mucinous adenocarcinoma	856 (9.2)	2580 (9.4)	.409	754 (11.8)	766 (8.3)	<.001
Chemotherapy						
Yes	5997 (64.1)	17,869 (65.4)		4466 (69.7)	6424 (69.5)	
No/unknown	3356 (35.9)	9461 (34.6)	.027	1940 (30.3)	2819 (30.5)	.774
T stage						
T1	77 (0.8)	769 (2.8)		12 (0.2)	182 (2)	
T2	303 (3.2)	2406 (8.8)		40 (0.6)	285 (3.1)	
T3	5698 (60.9)	18,220 (66.7)		2652 (41.4)	5171 (55.9)	
T4	3275 (35)	5935 (21.7)	<.001	3072 (57.8)	3605 (39)	<.001
N stage						
N0	0	0		0	2377 (25.7)	
N1	5547 (59.3)	19,075 (69.8)		2587 (40.4)	3333 (36.1)	
N2	3806 (40.7)	8255 (30.2)	<.001	3819 (59.6)	3533 (38.2)	<.001

### 3.1 Survival analysis

**FIGURE 2:**
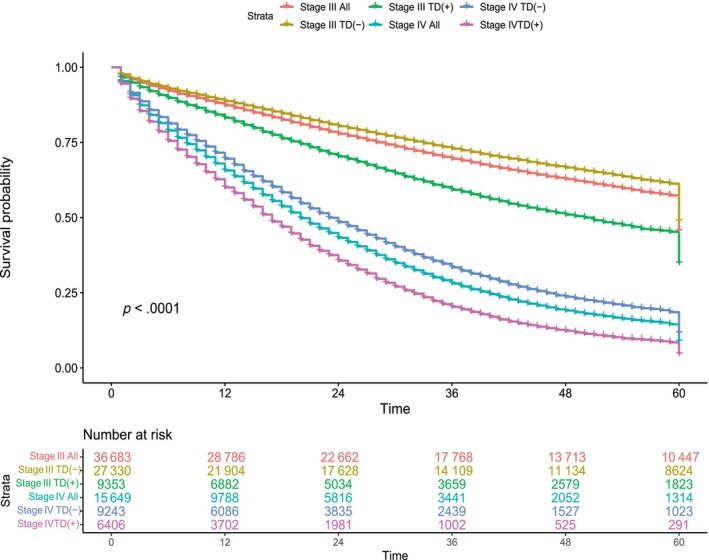
Five‐year OS of Stages III and IV patients. The Kaplan–Meier graph shows that patient with tumor deposits (TD) in both stages has a poorer prognosis. However, Stage III patients with TD still have significantly better survival compared to Stage IV patients.

In the overall Stages III and IV cohort, the 5‐year OS rates are 60% and 17%, respectively (Figure 2). In both stages, patients with TD exhibit significantly poorer prognosis compared to patients without TD, with 5‐year OS rates of 49% versus 64% for Stage III and 10% versus 21% for Stage IV, respectively (*p* < .001 per comparison).

### 3.2 Univariate and multivariate analysis

In both Stages III and IV patients, TD were associated with survival (Table 2). Multivariate analysis showed hazard ratios (HRs) of 1.4 (95% CI 1.4–1.5) and 1.3 (95% CI 1.2–1.3), respectively, when corrected for sex, age, tumor location, T and N categories, and treatment. Moreover, this analysis confirmed for both stages the value of TD in addition to these well‐known parameters.

**TABLE 2:** Univariate and multivariate Cox regression analysis of both stages.

	Stage III				Stage IV			
	Univariate analysis HR (95%)	*p*	Multivariate analysis HR (95%)	*p*	Univariate analysis HR (95%)	*p*	Multivariate analysis HR (95%)	*p*
Sex								
Female	1.0		1.0		1.0		1.0	
Male	0.996 (0.964–1.029)	.803	1.113 (1.077–1.150)	<.001	1.002 (.966–1.038	.933	1.057 (1.019–1.095)	.003
Age								
>65	1.0		1.0		1.0		1.0	
≤65	2.398 (2.313–2.485)	<.001	1.945 (1.873–2.020)	<.001	1.630 (1.573–1.689)	<.001	1.359 (1.309–1.410)	<.001
Tumor site								
Right colon	1.0		1.0		1.0		1.0	
Left colon	0.697 (0.674–0.722)	<.001	0.840 (.810–.870)	<.001	.691 (.666–.717)	<.001	.792 (.763–.823)	<.001
Large intestine	1.111 (.999–1.237)	.52	1.060 (.952–1.179)	.289	1.164 (1.046–1.295)	.005	1.112 (1.000–1.238)	.051
Tumor type								
Adenocarcinoma, nos	1.0		1.0		1.0		1.0	
Mucinous adenocarcinoma	1.174 (1.114–1.238)	<.001	1.049 (.995–1.106)	.079	1.170 (1.103–1.240)	<.001	1.007 (.949–1.068)	.825
Chemotherapy								
Yes	1.0		1.0		1.0		1.0	
No/unknown	2.744 (2.655–2.835)	<.001	2.446 (2.364–2.530)	<.001	2.825 (2.720–2.934)	<.001	2.758 (2.652–2.869)	<.001
T stage								
T1	1.0		1.0		1.0		1.0	
T2	1.365 (1.140–1.634)	<.001	1.219 (1.018–1.460)	.031	.529 (.428–.654)	<.001	.411 (.332–.509)	<.001
T3	2.127 (1.804–2.508)	<.001	1.744 (1.479–2.056)	<.001	.783 (.667–.920)	.003	.572 (.486–.673)	<.001
T4	3.799 (3.219–4.484)	<.001	2.972 (2.516–3.510)	<.001	1.123 (.956–1.320)	.157	.780 (.662–.918)	.003
N stage								
N0	—		—		1.0		1.0	
N1	1.0		1.0		1.224 (1.156–1.296)	<.001	1.250 (1.176–1.327)	<.001
N2	1.515 (1.466–1.567)	<.001	1.555 (1.503–1.609)	<.001	1.628 (1.541–1.720)	<.001	1.607 (1.514–1.707)	<.001
TD status								
Absent	1.0		1.0		1.0		1.0	
Present	1.547 (1.493–1.604)	<.001	1.419 (1.368–1.471)	<.001	1.416 (1.366–1.468)	<.001	1.275 (1.226–1.326)	<.001

Abbreviations: HR, hazard ratio; TD, tumor deposits.

## 4 DISCUSSION

In this retrospective SEER study, a comparison was conducted between the survival rates of Stage III colon carcinoma patients with TD and Stage IV patients. Our findings clearly indicate that although TD is associated with a significantly poor prognosis within Stage III category, their prognosis remains superior to that of Stage IV patients.

To the best of our knowledge, the idea of evaluating TD in M category was initially proposed by Puppa et al.^5^ In subsequent years, similar studies have been conducted, where patients with TD in Stage III exhibited poor survival and these results were interpreted as approaching to Stage IV prognosis.^6^, ^7^, ^8^ However, our findings diverge significantly from previous research. We observed a 5‐year OS rate of 17% for Stage IV patients, whereas Stage III patients with TD exhibited a 5‐year OS rate of 49%. The disparity in results might be the effect of small patient cohorts in these studies. The number of Stage III patients with TD varied between 22^8^ and 65.^5^ In this study, we have included 27.330 Stage III patients with TD. However, unrecorded and incomplete data variables in SEER, such as comorbidities, metastatic site, the intent of treatment (curative or palliative), and the lack of follow up details like recurrence status are the limitations of the survival analysis.^11^ The absence of histological review of TD samples in this study could be considered as another limitation, due to the poor reproducibility and ambiguous criteria for defining TD.^12^

Biological differences between Stages III and IV disease are clear. While in Stage III spread is still constrained to the locoregional lymph node draining areas of the colon, Stage IV disease is by definition systemic. Metastases to distant organs do not only threaten the function of those organs but can also cause systemic effects (paraneoplastic syndromes).^1^ It is true that in the presence of TD prognosis in all stages is relatively poor, as has been shown before for Stage III.^3^ We now confirm their prognostic impact in Stage IV disease as well, but regardless of the presence of TD in Stage IV patients, their outcome is significantly worse than that of Stage III CRC patients with TD.

Several scenarios have been proposed for the correct placement of TD in CRC staging.^3^ Our results imply that TD should be situated in the N category for locoregional spread. The current N1c category, in which TD are registered in the absence of LNM, is insufficient.^13^ The combination of LNM and TD shows significantly worse outcome, as has been shown before.^14^, ^15^ Several suggestions have been made for a more adequate inclusion of TD in nodal staging, including the simple add‐on method, also known as “counting principle” (i.e., count all TD as LNM) with a large number of supporting evidence and more complex subgroup designs.^14^, ^15^, ^16^, ^17^ For the latter, limited evidence is available, and its complexity will hamper clinical implementation.

## AUTHOR CONTRIBUTIONS

**Ayse Selcen Oguz Erdogan:** Data curation; formal analysis; investigation; visualization; writing—original draft. **Femke Simmer:** Conceptualization; methodology; supervision. **Iris D. Nagtegaal:** Conceptualization; funding acquisition; methodology; supervision.

## FUNDING INFORMATION

This study was funded by KWF Kankerbestrijding (KUN 2019‐12640): *Tumor deposits: the origin of distant metastases*?

## CONFLICT OF INTEREST STATEMENT

The authors have no conflicts of interest to declare.

## Data Availability

This study is based on the SEER database (seer.cancer.gov). Further details and other data that support the findings of this study are available from the corresponding authors upon reasonable request.

## References

[1] Steeg PS . Tumor metastasis: mechanistic insights and clinical challenges. Nat Med. 2006;12(8):895‐904. doi:10.1038/nm1469 16892035

[2] WHO Classification of Tumours Editorial Board . Digestive Systems Tumours, WHO Classification of Tumours Series. 5th ed. Lyon; 2019.

[3] Delattre JF , Oguz Erdogan AS , Cohen R , et al. A comprehensive overview of tumour deposits in colorectal cancer: towards a next TNM classification. Cancer Treat Rev. 2022;103:102325. doi:10.1016/j.ctrv.2021.102325 34954486

[4] American Joint Committee On Cancer . Manual for Staging of Cancer. 7th ed. New York, NY: Springer. 2010. Accessed March 11, 2024.https://cancerstaging.org

[5] Puppa G , Maisonneuve P , Sonzogni A , et al. Pathological assessment of pericolonic tumor deposits in advanced colonic carcinoma: relevance to prognosis and tumor staging. Mod Pathol. 2007;20(8):843‐855. doi:10.1038/modpathol.3800791 17491597

[6] Al Sahaf O , Myers E , Jawad M , Browne TJ , Winter DC , Redmond HP . The prognostic significance of extramural deposits and extracapsular lymph node invasion in colon cancer. Dis Colon Rectum. 2011;54(8):982‐988. doi:10.1097/DCR.0b013e31821c4944 21730787

[7] Tong LL , Gao P , Wang ZN , et al. Is the seventh edition of the UICC/AJCC TNM staging system reasonable for patients with tumor deposits in colorectal cancer? Ann Surg. 2012;255(2):208‐213. doi:10.1097/SLA.0b013e31821ad8a2 21527844

[8] Lino‐Silva LS , Anchondo‐Núñez P , Chit‐Huerta A , et al. Stage I‐III colon cancer patients with tumor deposits behave similarly to stage IV patients. Cross‐section analysis of 392 patients. J Surg Oncol. 2019;120(2):300‐307. doi:10.1002/jso.25482 31017669

[9] Park HS , Lloyd S , Decker RH , Wilson LD , Yu JB . Overview of the surveillance, epidemiology, and end results database: evolution, data variables, and quality assurance. Curr Probl Cancer. 2012;36(4):183‐190. doi:10.1016/j.currproblcancer.2012.03.007 22481006

[10] Surveillance, Epidemiology, and End Results (SEER) Program . (www.seer.cancer.gov). SEER*Stat Database: incidence – SEER research data, 17 registries, Nov 2022 sub (2000–2020) – linked to county attributes – time dependent (1990–2021) income/rurality, 1969–2021 counties, National Cancer Institute, DCCPS, Surveillance Research Program, released April 2023, based on the November 2022 submission.

[11] Park HS , Lloyd S , Decker RH , Wilson LD , Yu JB . Limitations and biases of the surveillance, epidemiology, and end results database. Curr Probl Cancer. 2012;36(4):216‐224. doi:10.1016/j.currproblcancer.2012.03.011 22481009

[12] Lord A , Brown G , Abulafi M , et al. Histopathological diagnosis of tumour deposits in colorectal cancer: a Delphi consensus study. Histopathology. 2021;79(2):168‐175. doi:10.1111/his.14344 33511676

[13] Ueno H , Nagtegaal ID , Quirke P , Sugihara K , Ajioka Y . Tumor deposits in colorectal cancer: refining their definition in the TNM system. Ann Gastroenterol Surg. 2023;7(2):225‐235. doi:10.1002/ags3.12652 36998291 PMC10043773

[14] Song YX , Gao P , Wang ZN , et al. Can the tumor deposits be counted as metastatic lymph nodes in the UICC TNM staging system for colorectal cancer? PLoS One. 2012;7(3):e34087. doi:10.1371/journal.pone.0034087 22461900 PMC3312887

[15] Cohen R , Shi Q , Meyers J , et al. Combining tumor deposits with the number of lymph node metastases to improve the prognostic accuracy in stage III colon cancer: a post hoc analysis of the CALGB/SWOG 80702 phase III study (Alliance)*. Ann Oncol. 2021;32(10):1267‐1275. doi:10.1016/j.annonc.2021.07.009 34293461 PMC8719434

[16] Pricolo VE , Steingrimsson J , McDuffie TJ , McHale JM , McMillen B , Shparber M . Tumor deposits in stage III colon cancer: correlation with other histopathologic variables, prognostic value, and risk stratification‐time to consider “N2c”. Am J Clin Oncol. 2020;43(2):133‐138. doi:10.1097/COC.0000000000000645 31764018 PMC7004443

[17] Pei JP , Zhang CD , Liang Y , et al. A modified pathological N stage including status of tumor deposits in colorectal cancer with nodal metastasis. Front Oncol. 2020;10:548692. doi:10.3389/fonc.2020.548692 33262940 PMC7686583

